# The Role of Dietary Vitamins and Antioxidants in Preventing Colorectal Cancer: A Systematic Review

**DOI:** 10.7759/cureus.64277

**Published:** 2024-07-10

**Authors:** Mohammed Ajebli, Christopher R Meretsky, Mourad Akdad, Ayoub Amssayef, Morad Hebi

**Affiliations:** 1 Biology Sciences, Faculty of Sciences and Technologies, Euromed University of Fes, UEMF, Fes, MAR; 2 Surgery, St. George's University School of Medicine, Great River, USA; 3 Biology Sciences, Faculty of Sciences and Technologies, Moulay Ismail University, Errachidia, MAR; 4 Pharmacology, Euromed University of Fes, UEMF, Fes, MAR; 5 Laboratory of Pharmacology-Toxicology, Faculty of Medicine, Pharmacy and Dentistry, Sidi Mohamed Ben Abdellah University, Fes, MAR

**Keywords:** prevention, oncology, dietary, antioxidants, vitamins, colorectal cancer (crc)

## Abstract

The role of dietary vitamins and antioxidants in preventing colorectal cancer (CRC) is a significant area of research within nutritional oncology. However, the relationship between these nutrients and CRC prevention is complex and influenced by factors such as dosage, timing, and individual health status. This review aims to comprehensively analyze and synthesize the existing scientific literature on the potential role of dietary vitamins and antioxidants in preventing CRC. A comprehensive literature review was conducted by searching electronic databases to identify studies examining the prospected impacts of dietary vitamins and antioxidants on the prevention of CRC. According to the outcomes of this review, this research review shows a complex link between vitamins and CRC. While some vitamins such as B2, B6, and D seemed helpful, others such as A and E had mixed results. Vitamin C deficiency was even linked to worse outcomes in cancer patients. Overall, the studies suggest focusing on a balanced diet rich in various vitamins rather than relying solely on individual supplements to prevent CRC. On the other hand, the results of our review suggest that the relationship between antioxidant intake and CRC is more intricate than previously thought. Data from this review indicates that taking specific antioxidant supplements such as selenium and vitamin E does not seem to offer the same protection. This suggests that a balanced diet with a variety of antioxidants is more helpful than focusing on single supplements. While we did not observe a direct association, future studies could investigate how different types and combinations of antioxidants might influence CRC development. In conclusion, the present systematic review highlights the need for more research on the relationship between vitamins, antioxidants, and CRC. We need to understand how these nutrients affect both the survival of people with CRC and the prevention of the disease. This will help us determine the best ways to use vitamins and antioxidants in CRC management and prevention.

## Introduction and background

Colorectal cancer (CRC) poses a significant public health challenge globally, contributing to substantial morbidity and mortality. Although it has traditionally been viewed as a disease predominantly affecting older adults, there has been a concerning rise in early-onset CRC among younger individuals in recent years [1]. CRC ranks as the third most common malignancy globally, accounting for approximately 10% of all cancer cases [2]. This high incidence rate highlights its significant impact on global health [3]. It is projected that, in 2018, approximately 576,000 men and 521,000 women will be diagnosed with CRC in the world [4]. In 2020, nearly two million new cases of CRC and approximately one million deaths from CRC were reported, accounting for 10.7% and 9.5% of all new cancer cases and deaths globally, respectively [5]. Furthermore, CRC is the fourth leading cause of cancer-related deaths, underscoring its severe mortality rate. The prevalence and fatality associated with CRC emphasize the urgent need for effective prevention, early detection, and treatment strategies [6]. Given its substantial contribution to the global cancer burden, continued research and public health initiatives are essential to address and mitigate the impact of this prevalent and deadly disease. According to recent reports from the World Health Organization, several lifestyle factors contribute to the development of CRC, including high intake of processed meats, low consumption of fruits and vegetables, a sedentary lifestyle, obesity, smoking, and excessive alcohol consumption [7].

The role of dietary vitamins and antioxidants in preventing CRC is a major focus within nutritional oncology. Vitamins and antioxidants, which are plentiful in fruits, vegetables, and whole grains, are recognized for their capacity to combat oxidative stress, a key contributor to the onset of cancer. These compounds, whether ingested through diet or supplements, can interact with free radicals in the body, potentially neutralizing them and preventing cellular damage that may lead to cancer. However, the relationship between these nutrients and CRC prevention is complex, influenced by factors such as dosage, timing, and individual health status [8]. Some studies have suggested a protective effect, whereas others have indicated no benefit or even potential harm [9]. Thus, further research is essential to fully understand the impact of dietary vitamins and antioxidants on CRC prevention and to create evidence-based dietary recommendations.

The purpose of this review is to comprehensively analyze and synthesize existing literature on the potential role of dietary vitamins and antioxidants in the prevention of CRC. We aim to provide a clear understanding of the current state of research in this area, identify gaps in knowledge, and suggest directions for future studies. By systematically reviewing studies that have investigated the relationship between dietary vitamins, antioxidants, and CRC, we seek to clarify whether these nutrients can effectively prevent the development of CRC. The findings of this review could have significant implications for dietary guidelines and public health strategies aimed at cancer prevention.

## Review

Material and methods

**Figure 1:**
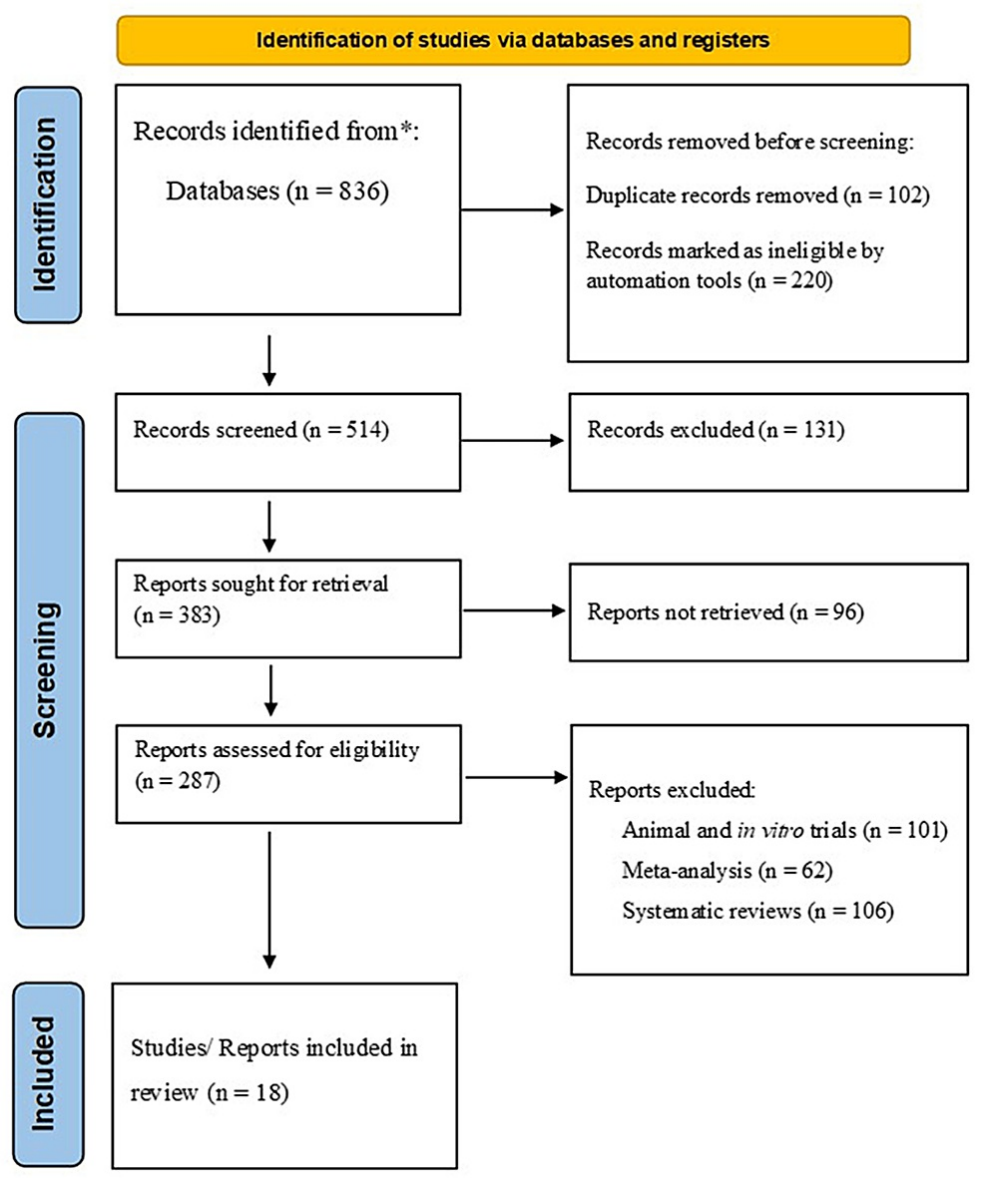
PRISMA diagram illustrating the study selection process. Following the PRISMA guidelines, our search was conducted using the academic databases PubMed, Medline, Google Scholar, ScienceDirect, and Springer with relevant keywords focused on the studies analyzing the anticipated effects of dietary vitamins and antioxidants on the prevention of CRC. We included studies published over a 10-year period (2014-2024) that met the inclusion criteria for this review paper [8]. Some records (n=131) may be excluded during the study selection process of this systematic review for various reasons, including 1) irrelevant study design and 2) irrelevant population (i.e., some records may focus on a population that is not relevant to the research question being addressed in the systematic review), 3) irrelevant intervention or outcome, and 4) poor quality (i.e., some records may be excluded due to methodological issues or poor study quality, which could lead to bias in the results of the systematic review). Some reports (96) may not be retrieved for our systematic review for various reasons, including 1) inaccessible full text, 2) language barriers, 3) publication bias, 4) time constraints, and 5) lack of resources. n: number; PRISMA: Preferred Reporting Items for Systematic Reviews and Meta-Analyses

We conducted a comprehensive review of the literature by searching electronic databases, including PubMed, ScienceDirect, Medline, Google Scholar, and Springer, to identify studies analyzing the anticipated effects of dietary vitamins and antioxidants on the prevention of CRC. We included recent studies published between 2014 and 2024 to capture the latest developments and trends regarding the protective role of these micronutrients against CRC. We restricted our search to English-language articles involving human participants. The search was conducted using specific keywords such as "Vitamins and colorectal cancer", "Vitamin D and CRC", "Vitamin E and CRC", "Antioxidants and CRC", "Potential effect of vitamins and antioxidants and CRC", and "Dietary vitamins and antioxidants and cancer". Adhering to the Preferred Reporting Items for Systematic Reviews and Meta-Analyses (PRISMA) guidelines ensured transparency and reproducibility throughout the review process (Figure 1).

After collecting and evaluating the data, the research team set the study objectives, determined the analysis methods, and established the inclusion and exclusion criteria. This sequence, however, is not recommended because it could introduce bias, thereby undermining the validity of the study. To maintain a robust, unbiased research framework and avoid limiting the applicability of the results, it is advisable to define these elements before data collection.

Study selection criteria and process

Inclusion Criteria

The inclusion criteria for this systematic review were randomized controlled trials, case reports, case studies, cohort studies, and technical notes published within the past 10 years (2014-2024) that investigated the prospective effect of vitamins and antioxidants on the prevention of CRC. To ensure the relevance and timeliness of the findings, we only considered studies from the past decade. The selected study types provided a comprehensive range of evidence, from high-quality randomized trials to observational studies and case reports, allowing for a thorough examination of the relationship between vitamin and antioxidant intake and CRC risk.

Exclusion Criteria

In this research, we have excluded studies that involve animals, *in vitro* assays, meta-analyses, systematic reviews, or retrospective analyses. We have also excluded abstracts of randomized controlled trials, case reports, case studies, cohort studies, or technical notes that investigate the effects of dietary vitamins and antioxidants on CRC prevention. Additionally, we have excluded any papers that do not focus on the effects of dietary vitamins and antioxidants on CRC prevention. We have only considered papers published in English and after 2014.

Results

Table 1 serves as a comprehensive repository of the most recent studies and trials exploring the potential influence of dietary vitamins in the prevention of CRC. The studies encapsulated in this table span a variety of research designs, including randomized controlled trials, observational studies, case-control studies, and cohorts derived from the Nurses’ Health Study (NHS) and the Health Professionals Follow-Up Study (HPFS).

**Table 1:** Studies from the last 10 years (2014-2024) have focused on analyzing the preventive effects of various vitamins on CRC. D3: Vitamin D3; VD: Vitamin D; VA: Vitamin A; VE: Vitamin E; VB: Vitamin B, VC: Vitamin C; VB2: Vitamin B2; VB6: Vitamin B6; VB12: Vitamin B12

Study Design	Vitamin	Cosupplementation	Participants	Interventions/Method	Follow-Up Period	Main Results	Conclusion	References
Randomized, double-blind, placebo-controlled trial	D3	Calcium	2,259 patients with recently diagnosed adenomas	Daily supplementation with VD3 (1000 IU), calcium carbonate (1200 mg), both, or neither	3 to 5 years	Mean net increase in serum 25-hydroxyvitamin D levels of 7.83 ng per milliliter; adjusted risk ratio for recurrent adenomas: 0.99 (95% CI, 0.89 to 1.09)	Daily supplementation with VD3 (1,000 IU), calcium (1,200 mg), or both after removal of colorectal adenomas did not significantly reduce the risk of recurrent colorectal adenomas over a period of 3 to 5 years	[9]
Randomized, double-blind, placebo-controlled trial	D3	Calcium	41 candidate single-nucleotide polymorphisms (SNPs) in 2,259 participants	Daily oral supplementation with vitamin D3 (1,000 IU) or calcium carbonate (1,200 mg elemental calcium), both, or neither		The impact of VD3 supplementation was observed on advanced adenomas, but not on the overall risk of adenomas For rs7968585, VD3 supplementation led to a 64% reduction in risk among individuals with the AA genotype. For individuals carrying one or two G alleles, VD3 supplementation resulted in a 41% increase in risk.	The effectiveness of VD3 supplementation in preventing advanced colorectal adenomas may depend on the individual’s VD receptor genotype.	[10]
Randomized, double-blind, placebo-controlled trial	D3	Calcium	2259 subjects were randomized to VD3	1,000 IU of vitamin D3 daily	3 or 5 years after the baseline exam	About 93% of subjects had a follow-up colonoscopy at least 1 year after randomization. Subjects given VD had levels 7.8 ng/ml higher than those given a placebo. During follow-up, 42% of subjects had one or more adenomas. The study treatments had no effect on adenoma outcomes, with relative risks (RR) for VD vs. placebo.	This study determined that additional intake of VD and calcium did not lower the risk of recurring colorectal adenomas over a period of 3 to 5 years.	[11]
Randomized, double-blind, placebo-controlled trial	D3	Omega-3 fatty acids	81 colorectal cancer patients	(1) Control group: receiving a VD3 placebo weekly + omega-3 fatty acid placebo capsules daily (2) Omega-3 fatty acid group: receiving 2 omega-3 fatty acid capsules daily + a VD3 placebo weekly (3) VD group: receiving a 50,000 IU VD3 weekly + 2 omega-3 fatty acid placebo capsules daily (4) cosupplementation group: receiving a 50,000 IU VD3 soft gel weekly + 2 omega-3 fatty acids capsules daily	8 weeks	There was a notable reduction in CRP and TNF-α levels in patients who were given a combination of VD3 and omega-3 fatty acids supplements, compared to those in the omega-3, VD3, and placebo groups. There was a significant decrease in the serum level of IL-6 in the omega-3, VD3, and combined supplementation groups compared to their initial levels. In terms of nutritional status, there was a significant increase in weight, BMI, and FFM% in the VD3, omega-3, and combined supplementation groups by the end of the intervention.	The combined supplementation of VD3 and omega-3 fatty acids in CRC patients has positive effects on inflammation and nutritional status.	[12]
A case–control study	VA and VE	Non	535 cases and 552 sex and age-matched controls	Face-to-face interviews using a validated food frequency questionnaire	5-year interval	Increased consumption of VA and VE was found to be associated with a 52% and 43% reduction in the risk of CRC, respectively. The study did not find a statistically significant relationship between serum levels of α-tocopherol and the risk of CRC. Higher serum levels of and higher dietary intake of VA and VE were associated with a reduced risk of CRC in both men and women.	This study provided evidence supporting the hypothesis that lower serum concentrations of retinol (a form of VA) and lower dietary intakes of VA and VE were associated with a decreased risk of CRC in a Chinese population.	[13]
A case–control study	VE and VD	Non	27,635 participants, among whom 183 individuals (0.6%) were diagnosed with CRC	Survey questionnaire data	2007–2018	VE intake was not associated with the development of CRC. The intake of total VD was also not related to the likelihood of developing CRC. There was no significant interaction or impact on these relationships.	The study found that the intake of both VD and VE were not associated with the occurrence of CRC.	[14]
Observational study	VC	None	Adults with metastatic colorectal cancer (mCRC; 46) and cancer-free controls (45)	-	-	VC intake was not significantly different between the two groups. Nonetheless, the mean plasma VC level was lower in the cancer group. The cancer group with VC deficiency showed a nonsignificant tendency toward higher mortality. Progression-free survival did not vary based on the presence of VC deficiency. Additionally, patients with BRAF and KRAS mutations did not exhibit significant differences in VC levels.	Patients with mCRC were found to have lower plasma VC levels compared to healthy control individuals. Although there was a trend toward higher mortality in the cancer group with VC deficiency, this difference was not statistically significant. Further exploration in phase III clinical trials is warranted to determine whether this phenomenon has an impact on patient survival and treatment response.	[15]
A case–control study	VB6	None	114,679: 613 CRC cases and 1190 matched controls nested	PLP, pyridoxal, pyridoxic acid (PA), 3-hydroxykynurenine, and xanthurenic acids (XAs) were measured in plasma with the use of HPLC/TMS	1985–2009	PLP concentrations were associated with a reduced risk of CRC for participants in the third quartile compared to those in the first quartile. Higher levels of the biomarkers HK:XA and PAr were associated with an increased CRC risk for participants in the fourth quartile compared to those in the first quartile.	Low levels of VB6, measured by the plasma concentration of PLP, are linked to a significant increase in the risk of CRC. VB6 may play a role in the progression of tumors rather than their initial development.	[16]
NHS and HPFS cohorts	VB2	None	3,037 incident CRC cases (2,093 women and 944 men)	Food frequency questionnaire	Every 4 years for 24–26 years	Intakes of total, dietary, and supplemental VB2 were inversely related to CRC risk in age-adjusted analysis in NHS.	The data from this prospective study do not provide evidence that higher intake of VB2 is associated with a reduced incidence or risk of CRC.	[17]
Observational study	VB12	Non	200 healthy adults	-	April–May 2013	A higher intake of VB12 is positively associated with a low-risk diet and lifestyle. Specifically, the intake of VB12 from milk, dairy products, and fish represents independent factors that contribute to a low-risk diet and lifestyle in this population at high-risk for CRC.	In a population at high risk for CRC, a higher intake of VB12, particularly from milk, dairy products, and fish, is associated with a low-risk diet and lifestyle.	[18]
A case–control study	VB (folate, B2, B6, and B12)	Methionine	2502 patients with CRC	Dietary data were collected using a validated FFQ	2010–2019	The risk of CRC decreased as the intake of folate and vitamins B2, B6, and B12 increased. There was no link found between methionine intake and the risk of CRC.	This research suggests that increasing the intake of folate, VB2, VB6, and VB12 may be linked to a lower risk of CRC in Chinese individuals.	[19]
A case–control study	VE	Non	975 cases and 975 age- and sex-matched controls	Dietary VE density (mg per 1,000 kcal) was assessed with a semiquantitative food frequency questionnaire.	CRC Patients: (1) 2010–2013; (2) 2018–2020 Control participant: 2007–2021	A higher VE density was linked to a reduced risk of CRC. When stratified by the COMT SNP rs740603 genotype, the inverse relationship between VE density and CRC risk was observed only in individuals carrying at least one A allele.	The findings suggest that a genetic polymorphism in COMT may influence the relationship between dietary VE intake and CRC risk.	[20]

Table 1 is accurately organized to present information on several key aspects of each study. This includes the specific vitamin under investigation, the use of cosupplementation (if applicable), the demographic targeted by the study, the intervention strategy or methodology employed, the duration of the follow-up period, and the principal outcomes and conclusions drawn from the study. This holistic summary facilitates effortless comparison across different studies, potentially aiding researchers in pinpointing areas ripe for future exploration and investigation. By collating a diverse array of studies from various sources, each employing different methodologies, Table 1 provides a panoramic view of the current landscape of research into the role of dietary vitamins in CRC prevention. This comprehensive approach underscores the table’s value as a vital resource for those engaged in this important area of research.

Vitamins

As illustrated in Table 1, various vitamins, including D, C, E, A, and B (specifically B6, B2, and B12), have been the focus of certain scientific investigations over the past decade (2014-2024). These studies encompassed randomized controlled trials, observational studies, and case-control studies and involved cohorts from the NHS and the HPFS.

Among these studies, four utilized a cosupplementation approach, where micronutrients were administered alongside the primary vitamin of interest. In all these cases, vitamin D (VD) was the central focus, cosupplemented with either calcium or omega-3 fatty acids to assess potential synergistic effects. Additionally, two studies employed a dual-vitamin approach, examining the combined effects of two vitamins administered simultaneously. One study investigated the interplay between vitamin A (VA) and vitamin E (VE), whereas the other explored the combined impact of VE and VD. This dual-vitamin approach provides valuable insights into the synergistic effects of vitamin combinations on health outcomes. This comprehensive body of research underscores the crucial role these vitamins play in human health and disease prevention. It highlights the importance of continued exploration into the synergistic and individual effects of vitamins, paving the way for future advancements in nutritional science and public health strategies.

Vitamin D

Although the potential benefits of VD supplementation have been widely studied, its impact on the recurrence of colorectal adenomas has not been found to be significant. Despite numerous trials and studies, the administration of VD supplements did not yield a substantial reduction in the risk of recurrent colorectal adenomas. This suggests that VD alone may not be sufficient to counteract the factors that contribute to the development of these precancerous growths. However, a different picture emerges when vitamin D3 (VD3) is combined with omega-3 fatty acids. In patients diagnosed with CRC, this combined supplementation has demonstrated positive effects. Specifically, it has been observed to improve inflammation markers and nutritional status. This is particularly noteworthy because inflammation is a known risk factor for many types of cancer, including CRC. Improvements in nutritional status can also contribute to overall health and well-being, potentially aiding in the recovery and management of the disease. Furthermore, the effectiveness of VD3 supplementation in preventing advanced colorectal adenomas appears to be influenced by genetic factors. More specifically, it may depend on the individual’s VD receptor genotype. This suggests that the benefits of VD3 supplementation could be more pronounced in individuals with certain genetic profiles. It underscores the importance of personalized medicine and the need to consider individual genetic variations when determining the most effective treatment and prevention strategies. Although vitamin D supplementation alone may not significantly reduce the risk of recurrent colorectal adenomas, its combination with omega-3 fatty acids and consideration of individual genetic factors could provide more promising results. Further research is needed to fully understand these relationships and to optimize supplementation strategies for CRC patients.

Vitamins A and E

Elevated serum levels and dietary intake of VA and VE have been found to be associated with a decreased risk of CRC in men and women. This suggests that these vitamins may play a protective role in the development of CRC. The protective effect could be due to the antioxidant properties of these vitamins, which help neutralize harmful free radicals in the body that can damage cells and lead to cancer. However, the relationship between VE intake alone and the development of CRC is not as straightforward. Although some studies have found a link, others have not, indicating that the relationship may be complex and influenced by other factors. It is important to note that, although VE is an essential nutrient, its role in cancer prevention is not yet fully understood, and more research is needed to clarify this relationship. Interestingly, a higher density of VE in the diet, which could be achieved by consuming foods rich in this vitamin, was associated with a reduced risk of CRC. This suggests that not only the amount but also the concentration of VE in the diet could be important for its protective effect against CRC. In addition to these findings, research has suggested that a genetic polymorphism in the catechol-O-methyltransferase (COMT) gene may influence the relationship between dietary VE intake and CRC risk. The COMT gene is involved in the metabolism of certain chemicals in the body, and variations in this gene could affect how the body processes and utilizes VE. This highlights the potential importance of genetic factors in determining individual responses to dietary interventions and the need for personalized nutrition strategies in cancer prevention. However, the role of VE in CRC prevention is complex and influenced by various factors, including genetic variations, higher serum levels, and dietary intake of VA and VE, as well as a higher density of VE in the diet, which appear to be associated with a reduced risk of this disease. Further research is needed to fully understand these relationships and their implications for CRC prevention strategies.

Vitamin B

Research has indicated that low levels of vitamin B6, as measured by the plasma concentration of pyridoxal 5’-phosphate (PLP), are associated with a significant increase in the risk of CRC. This suggests that vitamin B6 may play a crucial role in colorectal health and that maintaining adequate levels of this vitamin could be important for preventing the development of CRC. The exact mechanisms through which vitamin B6 might influence CRC risk are not fully understood, but it is thought that it may be involved in DNA synthesis and repair as well as in the regulation of cellular proliferation and differentiation. In contrast, the data from this prospective review do not provide evidence that a higher intake of vitamin B2 (VB2), also known as riboflavin, is associated with a reduced incidence or risk of CRC. This indicates that, despite its role in various cellular processes, VB2 may not have a significant protective effect against CRC. However, it is important to note that this does not diminish the overall importance of VB2 for health because it is essential for energy production and the metabolism of fats, carbohydrates, and proteins. In a population at high risk for CRC, a higher intake of vitamin B12 (VB12) was found to be associated with a low-risk diet and lifestyle. This vitamin, which is particularly abundant in cow’s milk, dairy products, and fish, may therefore contribute to CRC prevention. VB12 is involved in DNA synthesis and regulation, as well as in the metabolism of amino acids and fatty acids. Its deficiency can lead to anemia and neurological disorders. Therefore, maintaining adequate levels of this vitamin could be beneficial not only for colorectal health but also for overall well-being. Therefore, although the roles of vitamins B6 and B12 in CRC prevention appear to be significant, the role of VB2 is less clear. Further research is needed to fully understand these relationships and to develop effective dietary strategies for CRC prevention.

Antioxidants

Table 2 encapsulates a decade-long series of decade-long studies that scrutinized the potential role of dietary antioxidants in thwarting CRC. The studies encompass a diverse range of research designs, including case-control studies, observational studies, and prospective cohorts. Notable among these are the HPFS and the NHS. The table meticulously details the specific antioxidants and cosupplements under investigation. These include vitamin C (VC); VE; VD; folate; selenium; and a variety of carotenoids (β-carotene, lutein, zeaxanthin, and lycopene). The influence of these nutrients was gauged using surrogate measures such as plasma or dietary levels of the respective vitamins and antioxidants.

**Table 2:** Studies from the past 10 years (2014-2024) have focused on analyzing the preventive effects of various antioxidants on CRC. NHS: Nurses’ Health Study, HPFS: Health Professionals Follow-Up Study

Study	Antioxidant (and a Cosupplement if Existing)	Surrogate Used	Primary Objective	Assessed Parameters/Methodology	Participants	Main Outcomes	RR/HR/ORQ for CRC Occurrence (Compared to Placebo)	p-Value	Recommendation/Conclusion	References
Randomized Trial	Selenium and VE	Colorectal cancer (CRC) occurrence	Measure effect of selenium and effect of VE supplementation on colorectal adenoma occurrence	Occurrence of adenomas was established based on the findings of endoscopy and pathology reports for these procedures	6,546	Adenoma Occurrence included intervention: 34.2% Without intervention: 35.7%	Selenium: RR: 0.96 (95% CI, 0.90–1.02), VE: RR: 1.03 (95% CI, 0.96–1.10)	Selenium: 0.194; VE: 0.38	Neither selenium nor VE supplementation can be recommended for colorectal adenoma prevention.	[21]
Observational study	VC, VE, and ß-carotene	CRC etiology	The relationship between high total antioxidant intake and the risk of CRC	TAC was assessed by the Trolox equivalent antioxidant capacity test	45,194 persons enrolled in five centers	There was no significant relationship observed between dietary total antioxidant capacity (TAC) and the incidence of CRC. Intakes of VC, VE, and ß-carotene were not significantly associated with CRC risk.	Highest category of TAC: HR: 0.63 (95% CI: 0.44–0.89); Lowest category of TAC: HR: 2.09 (95% CI: 1.19–3.66)	Highest category of TAC: 0.008; Lowest category of TAC: 0.007	Future studies are necessary to verify the differing impacts of high total antioxidant consumption on the risk of developing CRC versus rectal cancer.	[22]
A case-control study	TAC	CRC and colorectal adenomatous polyps (CAP)	Investigate the association TAC with the odds of CRC CAP	Dietary ferric-reducing antioxidant potential and oxygen radical absorbance capacity	130 cases with incident, histologically confirmed CRC; 134 cases with incident of CAP and 243 hospital-based controls	TAC was significantly dependent to CRC and CAP odds.	ORQ3-Q1 for CRC = 0.25, 95% CI: 0.13–0.46, ORQ3-Q1 for CAP = 0.48, 95% CI: 0.27–0.85	CRC: 0.001 CAP: 0.01	The results of this study indicated an inverse relationship between TAC and the risk of CRC and coronary artery disease (CAD).	[23]
Observational study	Flavonoids and lignans	CRC	Assess the relationship between the dietary intake of flavonoids and lignans and the risk of CRC recurrence and overall survival in CRC patients.	Validated dietary questionnaires and lifestyle information were collected at recruitment	409 patients	After a mean follow-up period of 8.6 years, 32.5% had died, and 24.1% experienced a recurrence of CRC. The total intake of flavonoids was not associated with either the risk of CRC recurrence or overall survival in the multivariable analysis models.	HR for CRC recurrence: 1.13, 95% CI 0.64–2.02 HR for overall survival 1.06, 95% CI 0.69–1.65	CRC recurrence: 0.67 Overall survival: 0.78	No associations were observed between the intake of either total lignans or any specific flavonoid subclass and the risk of CRC recurrence or overall survival.	[24]
HPFS and NHS prospective cohorts	Flavonoid subclasses (flavonols, flavones, flavanones, flavan-3-ols, and anthocyanins)	CRC	Investigate the association between higher habitual dietary intake of different flavonoid subclasses and the risk of developing CRC.	Employing data gathered from validated food-frequency questionnaires administered every 4 years and an updated flavonoid food composition database, computed the flavonoid intakes for participants from the HPFS and NHS.	2519 colorectal cancer cases	The intake of different flavonoid subclasses was not associated with the risk of CRC in either of the studied cohorts. Further analysis by cancer subsite revealed that flavonoid intake was not associated with the risk of CRC or rectal cancer.	RRs (95% CIs) flavonols, 1.01 (0.89, 1.15) for flavones, 0.96 (0.84, 1.10) for flavanones, 1.07 (0.95, 1.21) for flavan-3-ols, and 0.98 (0.81, 1.19) for anthocyanins	All P-values for heterogeneity by sex >0.19	The results of the study do not provide evidence to support the hypothesis that a higher regular consumption of any flavonoid subgroup is associated with a reduced risk of developing CRC.	[25]
HPFS and NHS prospective cohorts	VC	CRC	Evaluate the relationship between VC intake and the survival of individuals with CRC, considering the presence or absence of KRAS or BRAF mutations in the tumor.	Used an inverse probability weighted multivariable Cox proportional hazards regression model to calculate the hazard ratio (HR) of mortality, and utilized spline analysis to assess the dose-response relationship between VC intake and CRC survival	2,096 CRC cases, of which 703 cases had KRAS and BRAF mutation data	The association between total VC intake and CRC-specific mortality appeared to vary based on the mutation status of the KRAS or BRAF genes. Higher VC intake was associated with a suggestive decrease in CRC-specific mortality in individuals with wild-type KRAS and BRAF genes, whereas the association was less clear in cases with either KRAS or BRAF mutations.	(1) The association between total VC intake and CRC-specific mortality suggestively differed according to KRAS or BRAF mutation status: HR (95% CI), 1.07 (0.87-1.32); (2) in cases with either KRAS or BRAF mutant type: 0.74 (0.55-1.00).	(1): 0.04 (2): < 0.05)	The findings suggest that the observed associations are in line with the laboratory evidence, indicating a potential benefit of higher VC intake for CRC patients with KRAS or BRAF-mutated tumors.	[26]

The primary objectives of these studies were to unearth the associations between these dietary factors and the risk of developing CRC. The studies varied in scale, with participant numbers ranging from hundreds to tens of thousands. The main outcomes were centered on the risk of CRC occurrence in relation to the examined dietary factors. The statistical analyses offered crucial insights into the potential protective effects of these micronutrients.

Drawing from the findings summarized in Table 2, the researchers put forth recommendations or conclusions about the potential advantages of augmenting the dietary intake or supplementation of these vitamins and antioxidants for CRC prevention. These recommendations serve as a testament to the power of dietary modifications in disease prevention and underscore the importance of continued research in this field.

Table 2 presents a comprehensive analysis of the impact of dietary supplements and nutrients on the risk of CRC. The findings suggest that neither selenium nor VE supplementation can be recommended for the prevention of CRC. This is because of the lack of significant evidence supporting their effectiveness in reducing the risk of this condition. The study also examined the intake of VC, VE, and ß-carotene. However, no significant associations were found between the intake of these nutrients and the risk of CRC. This indicates that these nutrients may not play a substantial role in CRC prevention. Interestingly, the study did find an inverse relationship between the TAC and the risk of both CRC and CAD. This suggests that a diet rich in antioxidants may potentially reduce the risk of these diseases. The research also explored the impact of lignans and flavonoids on CRC. It was found that there were no associations between the intake of either total lignans or any specific flavonoid subclass and the risk of CRC recurrence or overall survival. This suggests that these compounds may not have a significant effect on the progression or prognosis of CRC. Furthermore, the study found no evidence to support the hypothesis that higher regular consumption of any flavonoid subgroup is associated with a reduced risk of developing CRC. This challenges the common belief that flavonoids, which are known for their antioxidant properties, could potentially protect against CRC. Evidently, the findings suggest that the observed associations align with laboratory evidence, indicating a potential benefit of higher VC intake for CRC patients with KRAS or BRAF-mutated tumors. This could open new avenues for personalized nutrition strategies in CRC management.

Although some dietary supplements and nutrients may not significantly influence CRC risk, the potential role of antioxidants and the benefits of VC for certain CRC patients warrant further investigation. It is important to note that these findings should be interpreted with caution, and more research is needed to confirm these associations.

Discussion

Phytochemicals, vitamins, and antioxidants play a crucial role in the prevention and treatment of cancer including CRC. These naturally occurring compounds found in plants and other dietary sources possess a range of health benefits, including the ability to combat cancer. Interestingly, phytochemicals have been extensively studied for their potential to suppress cancer cell growth, alleviate inflammation, and protect against DNA damage that can lead to the initiation and progression of CRC. Certain phytochemicals, such as flavonoids, carotenoids, and polyphenols, have shown promising results in inhibiting the proliferation of this type of cancer cells and slowing disease progression.

Vitamins such as vitamin C, vitamin E, and vitamin D also play a crucial role in overall health and cancer prevention. These vitamins possess antioxidant properties that can help shield cells from damage and mitigate inflammation, which is a known risk factor for the development of CRC. Antioxidants such as selenium and zinc can also assist in neutralizing harmful free radicals in the body. While dietary phytochemicals, vitamins, and antioxidants can provide supplemental support in the prevention and treatment of CRC, they should not be considered as a sole or replacement therapy. A comprehensive approach to reducing the risk of CRC and promoting overall health includes a balanced diet rich in fruits, vegetables, whole grains, and lean proteins, coupled with regular physical activity and maintaining a healthy weight.

For personalized guidance on integrating these beneficial nutrients into diet, it is advisable to consult with a healthcare provider or a qualified nutritionist. This ensures that dietary changes are tailored to your individual health needs and conditions, optimizing the potential benefits for CRC prevention and overall well-being.

Vitamins

Our analysis of the studies presented in Table 1 reveals a consistent pattern of positive effects associated with the intake of various vitamins, including E, B2, B6, B12, D and D3, and A. Notably, the trend suggests that VC deficiency may be linked to higher mortality rates in cancer patients. However, the intake of both VD and VE did not show a significant correlation with the occurrence of CRC. In contrast, lower dietary intakes of VA and VE were associated with a decreased risk of CRC. Furthermore, the combined supplementation of VD3 and omega-3 fatty acids in CRC patients has been found to have positive effects. Interestingly, additional intake of VD and calcium did not lower the risk of recurring CRC. Only one study has suggested that VB6 may play a role in the progression of CRC tumors. These findings highlight the complex interplay between vitamins and their potential impact on CRC risk and outcomes.

Vitamin D

Emerging preclinical and epidemiological studies have indicated that VD could potentially exhibit anticancer properties, particularly in patients diagnosed with CRC. This is believed to be because of the role of VD in regulating cell growth and differentiation, which are key processes involved in cancer development and progression [27]. In light of these findings, a randomized phase II trial was recently conducted to investigate the effects of VD3 supplementation in patients living with metastatic CRC. The results of this trial were promising, showing the potential benefits of VD3 supplementation in managing this disease. Patients who received VD3 supplementation exhibited certain positive outcomes, although the specifics of these outcomes were not detailed in the initial statement. Encouraged by these positive findings, researchers have initiated a phase III trial to further investigate the potential benefits of VD3 supplementation in a larger cohort of patients with mCRC. This trial aims to confirm the results of the phase II trial and to determine whether VD3 supplementation should be incorporated into the standard care regimen for patients with mCRC. It is important to note that, although these results are promising, more research is needed to fully understand the role of VD in cancer treatment and prevention. As always, patients should consult with their healthcare provider before starting any new treatment regimen. Interestingly, the relationship between VD status and CRC has been extensively studied in epidemiological research. These studies have examined not only the incidence of the disease but also the survival of patients. When it comes to surrogates or indicators, used to measure VD status, the evidence is strong for the association with plasma 25(OH)D concentration, but the evidence is less robust for VD intake. To better interpret the data, the strengths and limitations of these surrogates are discussed in the context of the study design [28].

Preclinical research and epidemiological studies have suggested that VD may possess anticancer properties, particularly against CRC. However, clinical trials have not yet conclusively demonstrated the beneficial effects of VD in cancer prevention or treatment [29]. However, outcomes of this review (provided in Table 1) show that numerous studies and trials have been conducted, yet the use of VD supplements has not significantly decreased the risk of recurring colorectal adenomas. This implies that VD alone might not be enough to combat the elements that lead to the formation of these precancerous growths. In contrast, a recent meta-analysis reported that consuming a diet high in VD has been linked to a 25% reduced risk of developing CRC. However, more large-scale, high-quality prospective studies are required to conclusively confirm this association [30]. Similarly, to determine if low circulating VD levels are associated with poor CRC survival, Vaughan-Shaw et al. [31] evaluated whether VD supplementation improves CRC survival outcomes. They conducted a systematic search of PubMed and Web of Science, focusing on randomized controlled trials that reported CRC mortality. Using random-effects meta-analysis models, they calculated estimates of survival benefit from supplementation. Despite variations in inclusion criteria, intervention dose, and outcomes, their meta-analysis found a 30% reduction in adverse CRC outcomes with VD supplementation (n = 815, HR = 0.70; 95% confidence interval; CI: 0.48-0.93). A beneficial effect was observed in trials with CRC patients (progression-free survival, HR = 0.65; 95% CI: 0.36-0.94), with a suggestive effect in incident CRC cases from population trials (CRC-specific survival, HR = 0.76; 95% CI: 0.39-1.13). No heterogeneity or publication bias was detected. The meta-analysis demonstrates a clinically meaningful benefit of VD supplementation on CRC survival outcomes [31].

Conversely, the scenario changes when VD3 is paired with omega-3 fatty acids. This combination has shown positive effects in patients diagnosed with CRC, specifically improving inflammation markers and nutritional status. This is especially significant because inflammation is a recognized risk factor for various types of cancer, including CRC. Enhancements in nutritional status can also contribute to overall health and well-being, potentially assisting in the disease’s recovery and management. Moreover, the efficacy of VD3 supplementation in preventing advanced colorectal adenomas seems to be influenced by genetic factors. It may be dependent on the individual’s VD receptor genotype. This indicates that the advantages of VD3 supplementation could be more noticeable in individuals with specific genetic profiles. It highlights the significance of personalized medicine and the necessity to consider individual genetic variations when determining the most effective treatment and prevention strategies. Although VD supplementation alone may not significantly reduce the risk of recurrent colorectal adenomas, its combination with omega-3 fatty acids and consideration of individual genetic factors could yield more promising outcomes. More research is required to fully comprehend these relationships and to optimize supplementation strategies for CRC patients.

The available evidence suggests that a diet rich in certain dietary micronutrients involved in DNA methylation, such as folate, methionine, VB6, and B12, as well as antioxidant nutrients such as selenium, VE, and VC, may help lower the risk of CRC. Furthermore, this protective effect against CRC can be achieved through dietary means alone, without the need for supplementation [32]. Evidently, several potential mechanisms have been proposed to explain the involvement of VD in CRC development. These include VD’s role in promoting apoptosis, inducing differentiation in colonic epithelial cells, and suppressing angiogenesis [33].

Vitamins E and A

The scientific understanding of the anticancer potential of VE has evolved, with a growing emphasis on the role of tocotrienols (TTs) beyond the traditional focus on tocopherols. Emerging evidence has suggested that specific forms of tocotrienols, namely, γ-TT and δ-TT, possess the highest anticancer activities among the VE family [34]. Similarly, researchers at Stanford University School of Medicine have reported that retinoic acid, a compound derived from VA in the body, plays a crucial role in suppressing CRC in both mice and humans [35].

Our findings indicate that elevated serum levels and dietary intake of VA and VE have been linked to a decreased risk of CRC in both men and women, suggesting a protective role due to their antioxidant properties. However, the relationship between VE alone and CRC is complex and influenced by various factors, with some studies showing a link and others not. A higher dietary density of VE, achieved by consuming vitamin-rich foods, also correlates with reduced CRC risk. Evidently, over the past three decades, the potential anticancer effects of VE have been an area of active research. Multiple clinical trials have been conducted to evaluate the anticancer properties of α-tocopherol, which is a major isoform of VE. These trials have been based on the hypothesis that many types of cancer are associated with intense oxidative stress [36]. The Iowa Women’s Health Study, a longitudinal research project, investigated the relationship between dietary factors and the risk of CRC among women in Iowa. The findings suggest that a higher intake of VE was associated with a reduced risk of CRC, particularly for women under age 65 [37].

Additionally, genetic polymorphism in the COMT gene may affect the relationship between dietary VE and CRC risk, underscoring the importance of genetic factors in individual responses to dietary interventions. Overall, although the role of VE in CRC prevention is intricate, higher serum levels and dietary intake of VA and VE appear beneficial, warranting further research to fully understand these relationships. In this respect, scientists investigated genes involved in VE metabolism to see if variations in these genes might influence CRC risk. They analyzed data from public databases and their own experiments to examine how these genes were expressed in both healthy and cancerous tissues. Their findings suggested that a specific variation (rs73227586) in the SCARB1 gene was linked to an increased risk of CRC in Chinese people, and this association seemed to hold true for Europeans as well. Additionally, they observed that SCARB1 was much more active (expressed at higher levels) in colorectal tumors compared to healthy tissue. Further analysis suggested that the specific variant (T allele) affected how SCARB1 interacted with another molecule (ELL2), potentially promoting cancer development. Overall, this study suggests that variations in genes related to VE metabolism, particularly SCARB1, may be valuable tools for predicting CRC risk [38].

Vitamin B

Additional research has suggested that enhancing folic acid (a B vitamin) consumption could potentially reduce the likelihood of developing CRC among individuals who consume alcohol at moderate or high levels [39]. However, recent findings have suggested that folic acid and VB12 supplementation may increase the risk of CRC [40]. A recent meta-analysis indicates a nonsignificant decrease in CRC risk associated with high levels of vitamin B6 intake. However, in dose-response analysis, the risk reduction is significant [41].

Our findings have reported that low levels of VB6, as indicated by PLP concentration, are significantly associated with an increased risk of CRC. This suggests that VB6 may be crucial for colorectal health, potentially through its roles in DNA synthesis, repair, and regulation of cellular proliferation and differentiation. However, the study did not find evidence that a higher intake of VB2 (riboflavin) reduces CRC risk, despite its essential functions in energy production and metabolism. Conversely, higher intake of VB12, particularly from milk, dairy products, and fish, was linked to a low-risk diet and lifestyle in populations at high risk for CRC. VB12 is vital for DNA synthesis, amino acid metabolism, and overall health, highlighting its potential role in CRC prevention. Thus, although VB6 and VB12 appear significant in CRC prevention, the role of VB2 remains unclear, necessitating further research to develop effective dietary strategies.

Recent research has provided evidence suggesting that elevated serum VB12 concentrations are associated with CRC [42]. Furthermore, findings from a cohort study based on observation indicate that the consumption of VB6 and riboflavin, through diet and supplements, was linked to a reduced risk of CRC in women after menopause. The correlation between B vitamin intake and the disease was especially pronounced in cases of regional disease and among women who drank alcohol sparingly. Our research offers fresh insights suggesting that the rise in folate intake during the initial phase of fortification might have been connected to a temporary surge in CRC risk [43].

The scientific community has yet to reach a consensus on the role of vitamins in preventing CRC. According to the outcomes of the present literature, various studies present conflicting evidence, making it challenging to definitively ascertain the impact of vitamins on CRC prevention. One aspect under consideration is the cosupplementation of vitamins with other micronutrients, such as minerals, antioxidants, amino acids, or fatty acids. The hypothesis is that these micronutrients, when taken in conjunction with vitamins, could potentially moderate the vitamins’ effect on CRC prevention. This moderation could occur for several reasons, such as enhanced vitamin absorption or synergistic interactions that boost the body’s defense mechanisms against CRC. In contrast, the impact of vitamins consumed in isolation might not be as significant, possibly because of the absence of other essential nutrients required for optimal vitamin function. However, more comprehensive and rigorous studies are needed to validate these hypotheses and to better understand the complex interplay between vitamins, micronutrients, and CRC prevention. This research will aid in formulating more effective dietary guidelines and interventions for CRC prevention. It is important to note that, although diet and nutrition play a crucial role in disease prevention, they are just one piece of the puzzle. Other factors such as genetics, lifestyle, and environmental exposures also contribute to CRC risk and should be considered.

Moderate and adequate intake of vitamins is generally beneficial for the prevention of CRC. It is essential to consider the appropriate dosing of these vitamins and consult healthcare professionals for personalized advice. Furthermore, the potential benefits of cosupplementing these vitamins with other micronutrients should not be overlooked. Other micronutrients can interact synergistically with vitamins, enhancing their absorption and efficacy. This cosupplementation approach could amplify the preventive effects against CRC. However, it is important to approach vitamin and micronutrient supplementation with caution and professional guidance because excessive intake or improper combinations may lead to adverse effects or diminish the potential benefits. Therefore, informed decisions regarding vitamin intake and cosupplementation, based on current scientific evidence and medical advice, are crucial for optimizing CRC prevention and overall health.

Antioxidants

Recent data have indicated that the balance between total oxidants and antioxidants in the serum (total oxidant/antioxidant status) is significantly elevated in patients with CRC, whereas it is decreased in healthy individuals. Additionally, smoking and drinking habits have a significant influence on these levels [44]. Researchers have found a positive correlation between the antioxidant activity of plants and their antiproliferative effects. This suggests that the antioxidant properties of certain plant-derived compounds may contribute to their potential ability to inhibit the growth and proliferation of cancer cells [45]. In addition, several plant-based compounds, including genistein, curcumin, epigallocatechin-3-gallate, resveratrol, indole-3-carbinol, and proanthocyanidin, have exhibited promising patterns that suggest antioxidant intake may enhance the prognosis for advanced CRC patients when used as an adjuvant therapy [46].

The current review provides a comprehensive analysis of the impact of various dietary supplements and nutrients on the risk of colorectal adenoma and CRC. The findings indicate that selenium and VE supplementation cannot be recommended for colorectal adenoma prevention because of insufficient evidence of their effectiveness. Similarly, no significant associations were found between the intake of VC, VE, and ß-carotene and the risk of CRC, suggesting these nutrients may not substantially contribute to CRC prevention. However, the study did find an inverse relationship between TAC and the risk of both CRC and CAD, implying that a diet rich in antioxidants could potentially reduce the risk of these diseases. The research also examined the effects of lignans and flavonoids on CRC, finding no associations between the intake of total lignans or specific flavonoid subclasses and the risk of CRC recurrence or overall survival. This suggests that these compounds may not significantly affect CRC progression or prognosis. Furthermore, no evidence was found to support the hypothesis that higher regular consumption of any flavonoid subgroup reduces the risk of developing CRC, challenging the belief that flavonoids’ antioxidant properties could protect against CRC. Interestingly, the findings indicate a potential benefit of higher VC intake for CRC patients with KRAS- or BRAF-mutated tumors, aligning with laboratory evidence and suggesting new avenues for personalized nutrition strategies in CRC management. In addition, specific antioxidant supplements such as selenium and VE may not be effective in preventing colorectal adenomas. The data also showed no link between dietary intake of total lignans or specific flavonoid subclasses and CRC risk. In simpler terms, consuming a variety of antioxidant-rich foods might be helpful, but specific supplements targeting individual antioxidants may not be as beneficial for preventing CRC.

Numerous studies have suggested a potential link between selenium intake and the prevention of CRC. Findings from several observational studies have indicated that individuals with higher levels of selenium in their blood or diet tend to have a lower risk of developing CRC compared to those with lower selenium levels. These observational data provide preliminary evidence supporting a potential protective effect of selenium against colorectal carcinogenesis [47]. Previous reports indicated that antioxidants, such as VA, VC, VE, selenium, and β-carotene, when used individually, in combination with other antioxidants, or in combination with other agents, have not been found to be effective in preventing the development of colorectal neoplasia (including CRC and precancerous lesions) in the general population [48]. In addition, because of its antioxidant properties, VE is anticipated to help prevent cancer development [49]. Supporting this hypothesis, numerous epidemiological studies have reported that higher VE intake is associated with a reduced risk of various types of cancer [50,51]. In the same context, recent findings on gene-environment interactions have provided a possible explanation for the inconsistent results regarding VE and cancer. These findings suggest that genetic variants related to oxidant enzyme activity may have confounded the observed association between VE intake and cancer risk [52].

Our findings do not corroborate the hypothesis that the intake of antioxidants has a significant role in improving or preventing the pathogenesis of CRC. These results could potentially pave the way for further research into the impact of antioxidants on the survival rates among CRC patients, as well as the potential preventive benefits of this class of micronutrients for individuals at high risk of CRC.

## Conclusions

Our data generally indicate that dietary intake of vitamins has beneficial effects on CRC prevention. However, the evidence regarding the impact of vitamins on CRC prevention is contradictory. Notably, the cosupplementation of vitamins with other micronutrients-such as minerals, antioxidants, amino acids, or fatty acids-appears to enhance the preventive effects on CRC compared to the intake of vitamins alone. This synergistic approach may improve vitamin absorption and efficacy, boosting the body’s defense mechanisms against CRC. It is also important to recognize that the negative effects of vitamin intake on CRC prevention are minimal and typically associated with high doses or improper use of these micronutrients. Ensuring moderate and adequate vitamin consumption is essential, and consulting healthcare professionals for personalized advice can optimize the benefits of vitamins for CRC prevention. Furthermore, attention should be paid to the appropriate cosupplementation of vitamins with other essential nutrients to maximize their protective effects against CRC. This balanced approach underscores the importance of dietary and nutritional strategies in cancer prevention and overall health. Although previous investigations have highlighted the potential benefits of antioxidants in preventing and managing CRC, our findings do not support the hypothesis that antioxidant intake is directly associated with improving or preventing CRC pathogenesis. In other words, although eating a diet rich in antioxidants might help prevent CRC, taking specific antioxidant supplements such as selenium and VE does not offer the same protection. This suggests that a balanced diet with a variety of antioxidants is more helpful than focusing on single supplements. This finding diverges from previous studies and suggests that the role of antioxidants in CRC may be more complex than initially thought. Our results warrant further exploration into the nuanced relationship between antioxidants and CRC. First, future research could focus on investigating the specific impact of different antioxidants on CRC survival rates. Understanding which antioxidants, if any, contribute to improved survival outcomes for CRC patients is crucial. Furthermore, our findings underscore the need to examine the preventive potential of specific antioxidants for individuals at high risk of developing CRC. This research could focus on identifying and characterizing the dietary patterns and antioxidant profiles associated with reduced CRC risk.

Ultimately, our study contributes to the growing body of evidence demonstrating the need for further research into the complex interplay between vitamins and antioxidants and CRC. By investigating the potential impact of specific vitamins and antioxidants on CRC survival rates and preventive measures, we can better understand the role of these micronutrients in CRC management and prevention. Furthermore, conducting clinical trials to assess the efficacy of vitamin and antioxidant supplementation in improving CRC survival rates will provide valuable insights into personalized treatment strategies for patients. By rigorously evaluating the impact of these dietary supplements on CRC outcomes, researchers can gain a deeper understanding of how to optimize supportive care and enhance the overall management of the disease. in this respect, exploring the synergy between specific vitamins, antioxidants, and traditional CRC therapies can pave the way for enhanced treatment protocols and more effective outcomes in CRC patients. Investigating the potential for these natural compounds to complement or potentiate conventional cancer treatments, such as chemotherapy and radiation therapy, can lead to the development of more comprehensive and personalized treatment approaches. In addition, investigating the mechanisms by which vitamins and antioxidants influence CRC development and progression can lead to the development of targeted prevention strategies and interventions to reduce the global burden of colorectal cancer. By elucidating the underlying biological pathways and molecular mechanisms by which these dietary factors exert their protective effects, researchers can design more effective prevention programs and identify high-risk individuals who may benefit the most from tailored interventions. In the same background, collaborating with dietitians, clinicians, and researchers to create evidence-based guidelines on the optimal intake of vitamins and antioxidants for CRC prevention and management can empower individuals to make informed dietary choices for better health outcomes. By establishing comprehensive, evidence-based recommendations, healthcare professionals can provide clear guidance to the public on the role of these essential nutrients in reducing the risk and improving the outcomes of colorectal cancer.
